# Methodological discordance between apical four-chamber and biplane Simpson’s method for left ventricular ejection fraction: a retrospective study of a credentialed echocardiographic dataset

**DOI:** 10.1186/s12872-026-05952-0

**Published:** 2026-05-09

**Authors:** Hasan Burak İşleyen, Sevil Tugrul Yavuz, Sercan Bulut, Fatih Kizkapan, Cevahir Alioglu, Necla Zeynep Eren, Ali Arda Sozen, Mahsa Khanmohammadi

**Affiliations:** 1 Department of Cardiology, Nişantaşı University Faculty of Medicine, Istanbul, Turkey; 2 Department of Cardiology, Başakşehir Çam and Sakura City Hospital, Istanbul, Turkey; 3 Department of Cardiology, Bağcılar Training and Research Hospital, Istanbul, Turkey; 4 Department of Cardiology, Bezmialem Vakıf University Faculty of Medicine Hospital, Istanbul, Turkey

**Keywords:** Echocardiography, Left ventricular ejection fraction, Simpson’s biplane method, Apical four-chamber, Heart failure, Methodological discordance

## Abstract

**Background:**

Left ventricular ejection fraction (LVEF) remains central to heart failure phenotyping and device-based decision-making, yet the degree to which apical four-chamber (A4C) and biplane Simpson measurements diverge at clinically actionable thresholds is not well defined.

**Methods:**

We analysed 1,022 unique algorithmically derived echocardiographic studies from 784 patients in the credentialed MIMIC-IV-ECHO-Ext-LVVOLUMES-A4C-ROI resource. Each study contained paired A4C and biplane volumetric labels derived from the same annotated DICOM sequence. Discordance was defined primarily at the HFrEF threshold (LVEF < 40%). Agreement was assessed with Bland–Altman analysis, and independent predictors were evaluated using multivariable logistic regression with cluster-robust standard errors.

**Results:**

LVEF discordance at the HFrEF threshold occurred in 48 of 1,022 studies (4.7%, 95% CI 3.5–6.2%). At the ICD threshold (LVEF < 35%), discordance was present in 32 studies (3.1%). In the prespecified borderline zone (A4C LVEF 35–45%; *n* = 81), discordance rose to 30.9% (95% CI 21.9–41.6%). Mean bias was 0.11%, but the 95% limits of agreement were wide (− 13.5% to + 13.7%). LV end-diastolic volume was the only independent predictor of discordance (OR 1.61 per SD, 95% CI 1.27–2.05; *p* = 0.0001), and this association persisted after adjustment for acquisition variables.

**Conclusions:**

Discordance between A4C and biplane Simpson LVEF is uncommon across an unselected cohort but becomes frequent near therapeutic cut-offs. LV dilatation is the dominant driver. These findings support continued preference for biplane quantification when the ventricle is enlarged or the measured LVEF falls near a treatment threshold.

**Supplementary Information:**

The online version contains supplementary material available at 10.1186/s12872-026-05952-0.

## Introduction

Left ventricular ejection fraction (LVEF) remains one of the most practical variables in daily cardiology because it still anchors heart failure classification, treatment escalation, and a wide range of downstream decisions [1, 2]. Recent guideline updates have not altered that central role, but they continue to emphasise that method matters, especially when measurements sit near therapeutic cut-offs [3]. For two-dimensional echocardiography, the ASE/EACVI chamber quantification recommendations continue to favour the biplane Simpson method over single-plane approaches because it samples ventricular geometry more completely [4].

That distinction is not academic. A modest shift in measured LVEF can move a patient across thresholds used for HFrEF labelling, follow-up intensity, and device eligibility. Earlier work comparing echocardiographic approaches has shown that agreement in absolute LV volumes and EF is imperfect and that more comprehensive geometric sampling generally improves measurement fidelity [5]. Sequential reproducibility studies have also shown that method-dependent variation is clinically relevant when serial decisions depend on small changes in EF [6].

The present dataset allowed us to examine that question under unusually controlled conditions. All LV volumes and EF values were derived from the same annotated DICOM sequences rather than from separate clinical tracings. This reduces reader-dependent noise and makes the remaining disagreement easier to interpret as geometric discordance between formulas. The study therefore serves as a reproducible benchmark for contemporary quantitative echocardiographic workflows.

Using the credentialed MIMIC-IV-ECHO-Ext-LVVOLUMES-A4C-ROI resource [7], linked to the broader MIMIC-IV data infrastructure [8], we aimed to quantify how often A4C and biplane Simpson classifications disagree at clinically important LVEF thresholds and to identify the study-level features associated with that disagreement. Because MIMIC-IV v3.1 is queryable through BigQuery, the echocardiographic cohort could also be linked to structured hospital and ICU data without bulk export of the full raw database [9].

## Methods

### Data source and study population

We used the MIMIC-IV-ECHO-Ext-LVVOLUMES-A4C-ROI dataset (version 1.0.0, PhysioNet; DOI: 10.13026/713s-z339), a credentialed de-identified resource derived from the MIMIC-IV-ECHO module. The PhysioNet platform provides the framework through which such physiologic resources are curated and shared [10]. The dataset contains 1,064 apical four-chamber DICOM video sequences from 809 patients, each sequence representing one echocardiographic study with annotated ROI masks and paired algorithmic volumetric labels, including LVEDV, LVESV, and LVEF by both A4C single-plane and biplane Simpson methods. Device-level acquisition metadata, including frame rate, video duration, and scanner model, were linked by study-level identifiers from the distributed manifest.

Structured metadata from the MIMIC-IV-ECHO-Ext-LVVOLUMES-A4C-ROI dataset were used to define the echocardiographic cohort and derive a unified study timestamp for each examination. The cohort was then linked within Google BigQuery to MIMIC-IV v3.1 hospital, ICU, and derived concept tables using de-identified subject identifiers, hospital admission identifiers, ICU stay identifiers, and prespecified temporal proximity rules. To avoid downloading the full raw database, all processing was performed directly in BigQuery using selective SQL-based extraction. Only predefined demographic, outcome, ICU, laboratory, hemodynamic, vasopressor, and severity variables were retrieved, and time-dependent measurements were summarised within a − 24 to + 24 h window around each echocardiographic study. The final output was a single structured analysis table integrating echo-derived labels with linked clinical variables. During revision, additional linked candidates such as height, weight, blood pressure, hyperlipidaemia, prior myocardial infarction, vasoactive support, and NYHA class were re-queried; however, completeness after study-level linkage was heterogeneous and NYHA yielded no usable structured records, so these fields were not promoted to the core comparative table. The selection process is summarised in Additional file 1.

No study-specific manual tracing was performed for the present analysis. We analysed pre-existing dataset labels that were derived from annotated ROI masks provided within the credentialed resource. Because the measurements were not newly generated by a study reader, blinding to the study hypothesis and inter- or intra-observer variability testing were not applicable to this secondary dataset analysis.

### Outcome definitions

The primary outcome was discordance in categorical classification at the HFrEF threshold, defined as disagreement between A4C and biplane Simpson classifications using LVEF < 40% versus ≥ 40%. Secondary outcomes were discordance at the ICD threshold (LVEF < 35%) and at the HFmrEF threshold (LVEF < 50%). A prespecified borderline zone of A4C LVEF 35–45% was chosen to bracket the 35% and 40% clinical decision bands. Sensitivity analyses repeated the borderline analysis using biplane LVEF 35–45%, mean LVEF 35–45%, and A4C LVEF 37.5–42.5% (Additional file 2). A representative A4C frame, the exact ROI mask, and an overlay example are provided in Supplementary Figure S2 (Additional file 3).

### Statistical analysis

Continuous variables are reported as mean ± SD. Categorical variables are presented as counts and percentages with 95% confidence intervals calculated using the Wilson/Newcombe approach for single proportions [11]. Group comparisons used independent-samples t-tests and chi-square tests.

Agreement between A4C and biplane LVEF was evaluated with Bland–Altman analysis, including confidence intervals for the mean bias and the limits of agreement, following the methodological recommendations of Bland and Altman [12]. Pearson correlation was reported descriptively and was not used as a substitute for agreement.

Because 169 of 784 patients contributed more than one study, logistic regression models were fitted with patient-level clustering and robust sandwich covariance estimation. This approach follows the classic Huber and White framework for robust inference in the presence of non-independence and model misspecification [13, 14]. The primary model included standardised LVEDV, age, and biological sex. A sensitivity model added frame rate, video duration, and device model. Discordance rates across LVEDV quartiles were compared using a chi-square test for trend. LVESV was not entered together with LVEDV in the primary multivariable model because both variables index chamber size from the same study and were expected to be strongly collinear; to preserve model stability with 48 discordant events, we retained LVEDV as the pre-specified ventricular size marker.

All analyses were performed in Python 3.x using NumPy, SciPy, and scikit-learn. Two-sided p values < 0.05 were considered statistically significant. Access to the underlying PhysioNet resource was obtained under approved credentialed access; required training was completed and the applicable data use agreement was fulfilled. The accessing author held PhysioNet credential record number 75,660,195.

## Results

### Study population

The analytic sample comprised 1,022 echocardiographic studies from 784 patients (Table 1). Of 784 patients, 615 (78.4%) contributed a single study; 169 (21.6%) contributed two or more (maximum 6; mean 1.30 ± 0.72). Mean age was 65.9 ± 12.8 years; 53.5% of studies were from female patients. Studies were acquired on GE platforms only. Additional linked clinical characteristics were directionally similar between concordant and discordant studies: hypertension 49.3% vs. 58.3% (*p* = 0.308), diabetes mellitus 24.0% vs. 33.3% (*p* = 0.242), atrial fibrillation 27.8% vs. 29.2% (*p* = 1.000), coronary artery disease 30.8% vs. 41.7% (*p* = 0.184), and recorded heart failure diagnosis 31.4% vs. 41.7% (*p* = 0.222). Body mass index was similar (29.5 ± 6.7 vs. 30.1 ± 7.8 kg/m²; *p* = 0.645).

**Table 1:** Baseline Characteristics of Concordant and Discordant Studies

Variable	Concordant (*n* = 974)	Discordant (*n* = 48)	*p*-value
Age (years), mean ± SD	66.0 ± 12.8	63.5 ± 13.2	0.201
Female sex, *n* (%)	521 (53.5%)	26 (54.2%)	0.926
Studies per patient, mean ± SD	1.30 ± 0.72	1.31 ± 0.73	0.944
LVEF A4C (%), mean ± SD	59.2 ± 13.5	39.0 ± 13.9	< 0.001
LVEF biplane (%), mean ± SD	59.2 ± 13.1	38.8 ± 12.7	< 0.001
|LVEF A4C−biplane| (%), mean ± SD	4.4 ± 3.8	11.3 ± 6.8	< 0.001
Hypertension, *n* (%)	480 (49.3%)	28 (58.3%)	0.308
Diabetes mellitus, *n* (%)	234 (24.0%)	16 (33.3%)	0.242
Atrial fibrillation, *n* (%)	271 (27.8%)	14 (29.2%)	1.000
Coronary artery disease, *n* (%)	300 (30.8%)	20 (41.7%)	0.184
Recorded heart failure diagnosis, *n* (%)	306 (31.4%)	20 (41.7%)	0.222
Body mass index (kg/m²), mean ± SD	29.5 ± 6.7	30.1 ± 7.8	0.645
LVEDV A4C (mL), mean ± SD	103 ± 50	148 ± 82	< 0.001
LVESV A4C (mL), mean ± SD	44 ± 34	87 ± 51	< 0.001
Vivid E95, *n* (%)	607 (62.3%)	27 (56.2%)	0.659
Vivid S70, *n* (%)	343 (35.2%)	20 (41.7%)	
Vivid E90, *n* (%)	24 (2.5%)	1 (2.1%)	

One DICOM sequence = one unique study. Discordance = disagreement in HFrEF classification (LVEF < 40%) between A4C and biplane methods. All LV values algorithmically derived from annotated ROI masks

### Bland–Altman agreement

**Fig. 1:**
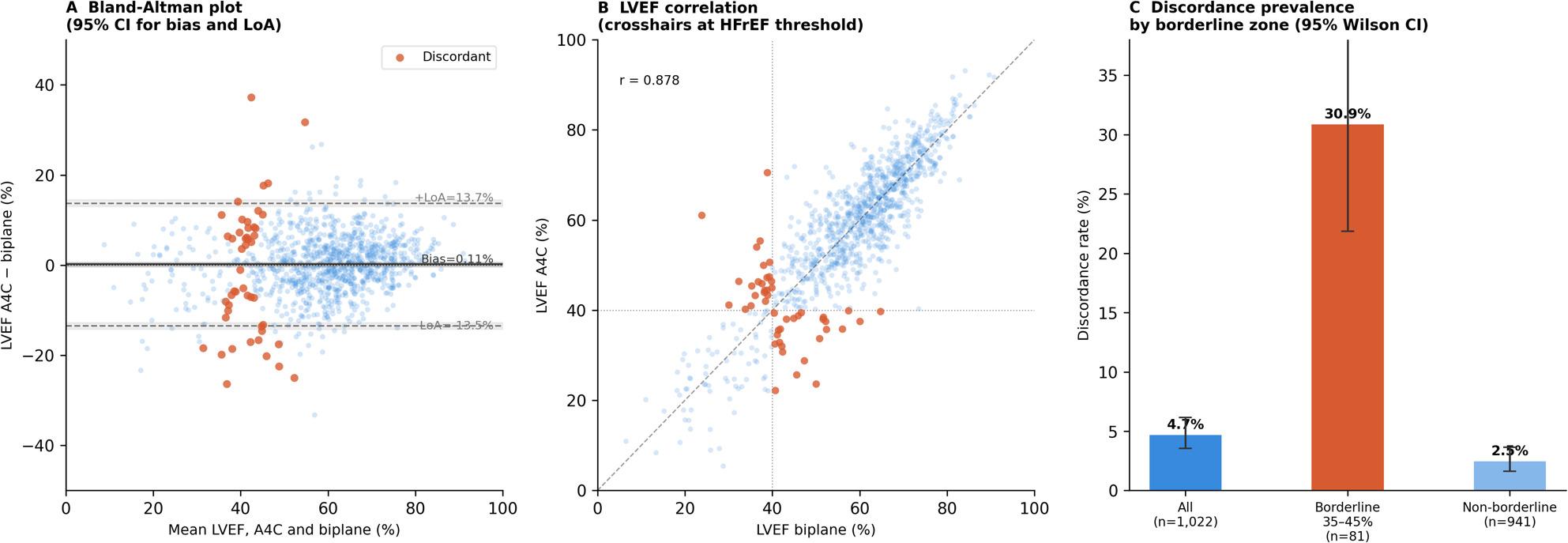
Agreement and threshold discordance between apical four-chamber (A4C) and biplane Simpson’s LVEF (n=1,022 unique studies from 784 patients). **A** Bland–Altman plot. Solid line: mean bias (0.11%); dashed lines: 95% limits of agreement; shaded bands: 95% CIs per Bland & Altman (1999). Discordant studies in coral. **B** LVEF scatter (A4C vs biplane); dashed crosshairs at the 40% HFrEF threshold. **C** Discordance in all studies, the prespecified borderline zone (A4C 35–45%, n=81), and the non-borderline population, with 95% Wilson CIs. A4C, apical four-chamber; HFrEF, heart failure with reduced ejection fraction; LVEF, left ventricular ejection fraction

Mean bias was 0.11% (95% CI: −0.31 to + 0.54%), confirming no systematic directional offset. The 95% limits of agreement were − 13.5% (95% CI: −14.2 to − 12.8%) to + 13.7% (95% CI: +13.0 to + 14.5%), reflecting a clinically wide inter-method spread (Fig. 1A; Table 2). Pearson *r* = 0.878 is reported descriptively (Fig. 1B).

**Table 2:** Bland–Altman Agreement and Classification Performance Relative to Biplane Reference

Metric	Value	95% CI
Pearson r (A4C vs. biplane)	0.878	0.869–0.887
Mean bias, A4C−biplane (%)	0.11	−0.31 to + 0.54
Upper limit of agreement (%)	13.7	13.0 to 14.5
Lower limit of agreement (%)	−13.5	−14.2 to − 12.8
Sensitivity (A4C for HFrEF)	76.0%	65.8–84.4%
Specificity	97.3%	96.2–98.1%
Positive predictive value	74.5%	64.4–82.9%
Negative predictive value	97.5%	96.5–98.3%
Discordance — HFrEF (< 40%)	4.7% (48/1,022)	3.5–6.2%
Discordance — ICD threshold (< 35%)	3.1% (32/1,022)	2.2–4.4%
Borderline zone (A4C 35–45%)	30.9% (25/81)	21.9–41.6%

Limits of agreement 95% CIs per Bland & Altman (1999). Wilson score 95% CIs for proportions

### Discordance prevalence and borderline zone

LVEF discordance at the HFrEF threshold was present in 48/1,022 studies (4.7%, 95% CI: 3.5–6.2%). Of these, 25 were A4C-lower discordant (A4C < 40%, biplane ≥ 40%) and 23 biplane-lower discordant, with no systematic directional bias. Discordance at the ICD threshold was present in 32/1,022 (3.1%). In the pre-specified borderline zone (A4C LVEF 35–45%; *n* = 81), discordance was 30.9% (95% CI: 21.9–41.6%), compared with 2.5% in the non-borderline population. The distribution of discordant threshold classification is further illustrated in Fig. 1C. Sensitivity analyses using alternative borderline definitions yielded consistent results: 34.5% (biplane 35–45%), 48.7% (mean LVEF 35–45%), and 37.5% (A4C 37.5–42.5%) — all substantially elevated relative to the overall discordance rate (Supplementary Table S1).

### Predictors of discordance

In multivariable logistic regression, older age, greater interventricular septal thickness, greater posterior wall thickness, and larger left ventricular end-diastolic volume (LVEDV) were independently associated with increased odds of threshold discordance, while higher biplane LVEF was inversely associated. Sex, heart rate, rhythm irregularity, mechanical ventilation, and vasopressor use were not independently associated after adjustment (Table 3).

**Table 3:** Multivariable Logistic Regression: Naive vs. Cluster-Robust Estimates

Variable	Naive OR (95% CI)	Naive *p*	Robust OR (95% CI)	Robust *p*
LVEDV (per SD)	1.61 (1.32–1.97)	< 0.001	1.61 (1.27–2.05)	0.0001
Age (per SD)	1.06 (0.80–1.40)	0.671	1.06 (0.78–1.43)	0.720
Sex (male vs. female)	0.92 (0.51–1.66)	0.775	0.92 (0.48–1.75)	0.796

Cluster-robust SE: Huber-White sandwich estimator, clustering unit = patient. Both approaches yield identical OR estimates; 95% CIs are marginally wider under the robust approach. Variables standardised (z-scored). *LVEDV * left ventricular end-diastolic volume

**Fig. 2:**
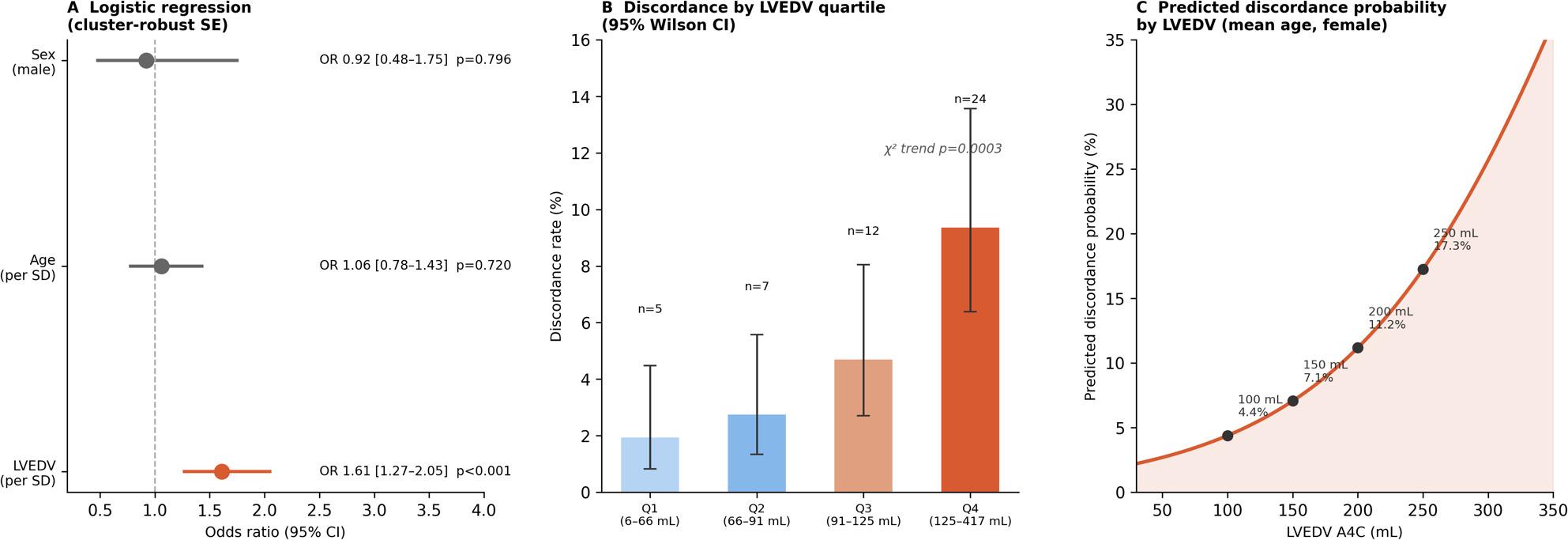
LV end-diastolic volume (LVEDV) as the main structural correlate of discordance. **A** Forest plot of multivariable logistic regression with cluster-robust standard errors. **B** Discordance by LVEDV quartile with 95% Wilson CI error bars; chi-square trend p=0.0003. **C** Model-estimated discordance probability across increasing LVEDV values at mean age and female sex. LVEDV, left ventricular end-diastolic volume

Sensitivity analyses that retained one randomly selected study per patient showed consistent findings (Supplementary Table S2). Additional sensitivity analyses adjusting for frame rate, video duration, and device model did not materially alter results (Supplementary Table S3). For clinical interpretability, we also fit scaled models using thresholds such as 5 years of age, 5 mm of wall thickness, 10 mL of LVEDV, and 1% of LVEF; larger wall thickness and chamber size remained associated with discordance, whereas absolute effect sizes per unit increment were smaller on the original scale (Supplementary Table S4). In supplementary linked analyses, septal thickness, posterior wall thickness, and LVEDV remained independently associated with discordance after additional adjustment for body size and image characteristics, although confidence intervals widened because of missing structural covariates and reduced sample size (Supplementary Table S5). Results were fully preserved in the one-study-per-patient sensitivity analysis (Supplementary Table S2). The cluster-robust regression estimates and the LVEDV quartile gradient are shown in Fig. 2; Table 4.

**Table 4:** Discordance Rate by LVEDV Quartile

LVEDV Quartile	Range (mL)	*n* studies	Discordant, *n* (%)	95% Wilson CI
Q1	6–66	257	5 (1.9%)	0.8–4.5%
Q2	66–91	254	7 (2.8%)	1.3–5.6%
Q3	91–125	255	12 (4.7%)	2.7–8.0%
Q4	125–417	256	24 (9.4%)	6.4–13.6%

Chi-square test for trend across quartiles: *p* = 0.0003

## Discussion

This study yields three clinically relevant observations. First, overall discordance between A4C and biplane Simpson classifications was not common in the full cohort, but it became frequent in the narrow range where decisions are most vulnerable to method drift. Second, a negligible mean bias coexisted with wide limits of agreement; those two findings are not contradictory, but rather describe different levels of comparison. A near-zero average difference can coexist with clinically relevant classification discordance because positive and negative individual deviations cancel in the mean, whereas threshold crossing is driven by the spread of individual differences. Third, LV dilatation was the only stable predictor of discordance. These findings are consistent with older comparisons showing that simplified geometric assumptions become less reliable as ventricular shape departs from a compact ellipsoid [15].

The signal was even clearer in the borderline zone. A patient with a measured LVEF in the mid-30s or low-40s does not live in the abstract world of summary bias; that patient lives in the world of threshold-based treatment. In modern automated three-dimensional workflows, reproducibility improves, but disagreement between simplified and more complete geometric representations does not disappear [16]. Recent outcome data have also suggested that more complete volumetric quantification can carry incremental prognostic information beyond conventional two-dimensional estimates [17].

The present analysis is deliberately narrower than a conventional clinical reproducibility study. It isolates the geometric component of disagreement by removing most operator-level tracing variability. In practice, the true clinical spread is likely to be wider, not narrower. That is precisely why this type of benchmark is useful: it identifies the minimum disagreement that remains even before acquisition quality, endocardial definition, and reader technique are allowed to vary.

The threshold implications are difficult to ignore. In the era that established ICD benefit in patients with marked LV systolic dysfunction, trial enrolment was anchored to low LVEF cut-offs [18, 19]. Our findings do not challenge those trials; rather, they remind us that the measurement method used to determine eligibility still matters. When the ventricle is enlarged or the measured LVEF falls in the 35–45% range, biplane quantification should be preferred over a single-plane estimate, and persistent uncertainty should prompt consideration of three-dimensional echocardiography or cardiac magnetic resonance. This practical emphasis is aligned with broader contemporary echocardiographic methodology statements [20].

### Limitations

Several limitations deserve emphasis. All LV measurements were algorithmically derived from annotated ROI masks rather than prospectively generated clinical tracings. The data originated from a single healthcare system and from GE platforms only, which may narrow technical generalisability. Importantly, because this was a secondary analysis of a structured dataset, study-specific observer variability could not be tested and structural markers such as LVPW or LV mass were not available in usable form for the full cohort. The deliberately controlled design also means that our estimates likely represent a lower bound of real-world disagreement, because acquisition heterogeneity and reader-level tracing variability were largely removed.

## Conclusion

Discordance between A4C and biplane Simpson LVEF is modest in an unselected credentialed echocardiographic cohort but becomes clinically meaningful near treatment thresholds. LV dilatation consistently marks the subgroup at greatest risk of crossing a categorical boundary. In day-to-day practice, these results support a cautious preference for biplane quantification when the ventricle is enlarged or the measured LVEF falls near the zone in which therapy may change; reporting systems may also benefit from flagging large A4C-versus-biplane discrepancies for method reconciliation.

## Supplementary Information

Supplementary Material 1.

Supplementary Material 2.

Supplementary Material 3.

## Data Availability

The underlying PhysioNet dataset is available under credentialed access and is not redistributed by the authors. Researchers must obtain their own approved access through PhysioNet. The public analysis repository contains code, documentation, and supplementary materials, but no credentialed raw data [21].

## Abbreviations

A4C: Apical four-chamber
EF: Ejection fraction
HFrEF: Heart failure with reduced ejection fraction
HFmrEF: Heart failure with mildly reduced ejection fraction
ICD: Implantable cardioverter-defibrillator
LV: Left ventricle/left ventricular
LVEDV: Left ventricular end-diastolic volume
LVESV: Left ventricular end-systolic volume
