# Supplementary figures and images for: Methodological discordance between apical four-chamber and biplane Simpson’s method for left ventricular ejection fraction: a retrospective study of a credentialed echocardiographic dataset
Source: BMC Cardiovasc Disord. 2026 May 9;26:561. doi: 10.1186/s12872-026-05952-0 (PMC13330419; doi:10.1186/s12872-026-05952-0)

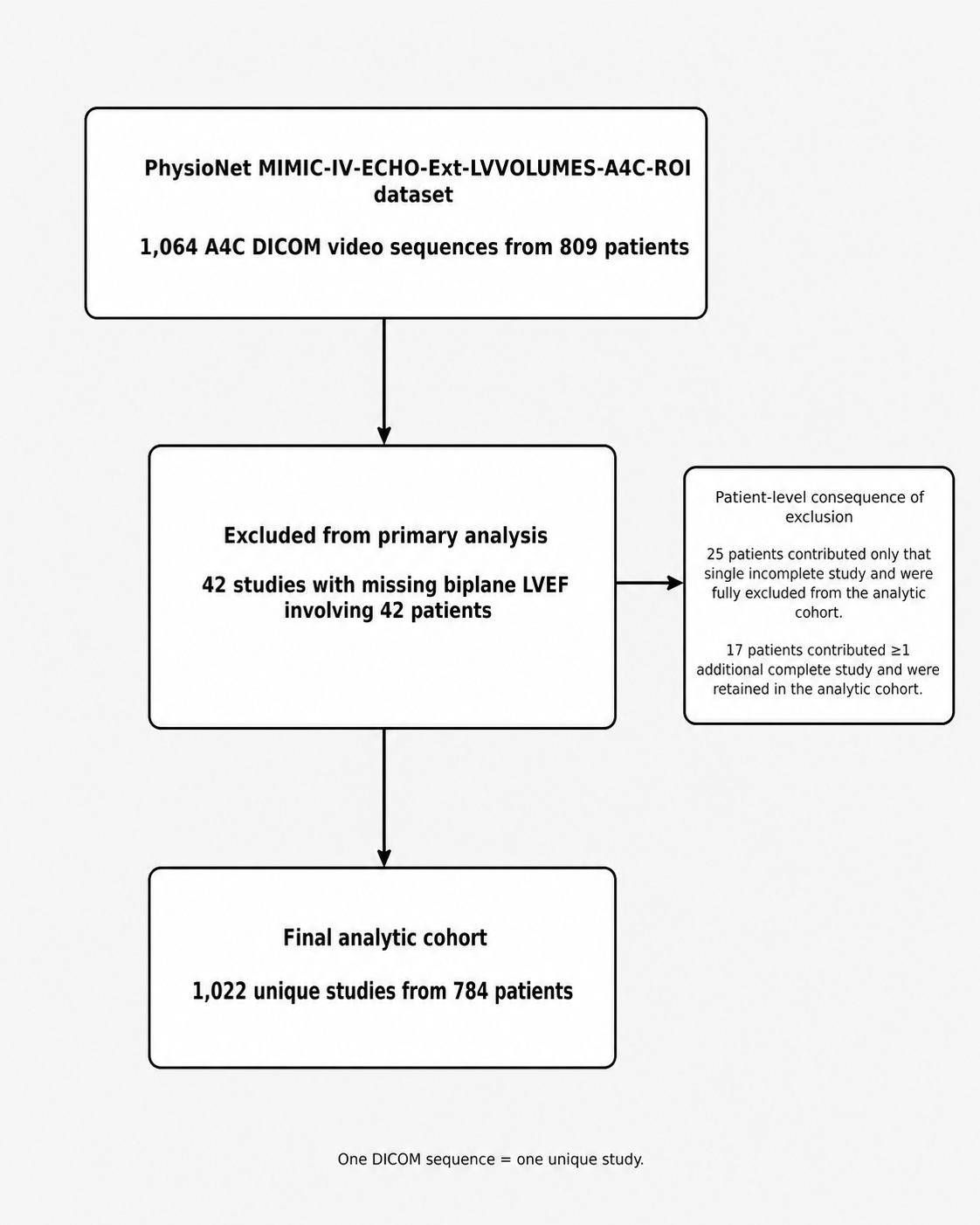

Supplement: Supplementary file 1 — Supplementary Material 1. [file 12872_2026_5952_MOESM1_ESM.png]

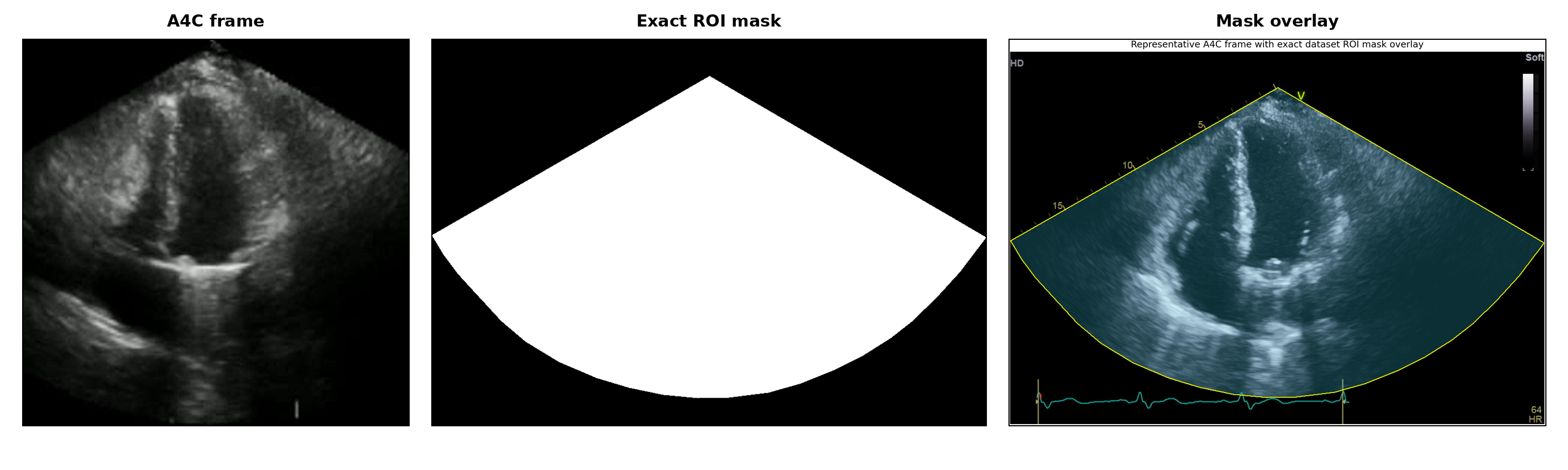

Supplement: Supplementary file 3 — Supplementary Material 3. [file 12872_2026_5952_MOESM3_ESM.png]
