# Supplementary material for: Methodological discordance between apical four-chamber and biplane Simpson’s method for left ventricular ejection fraction: a retrospective study of a credentialed echocardiographic dataset
Source: BMC Cardiovasc Disord. 2026 May 9;26:561. doi: 10.1186/s12872-026-05952-0 (PMC13330419; doi:10.1186/s12872-026-05952-0)

**Supplementary Appendix**

Manuscript title: Methodological Discordance Between Apical Four-Chamber and Biplane Simpson’s Method for Left Ventricular Ejection Fraction: a retrospective study of a credentialed echocardiographic dataset

**Supplementary Table S1. Alternative borderline-zone definitions and discordance yield**

Borderline zone analyses using neighbouring A4C thresholds showed the same pattern as the primary 35–45% definition: discordance was concentrated around decision-adjacent values and remained low outside that range.

**Supplementary Table S2. Sensitivity multivariable model including acquisition variables**

| Predictor | OR | 95% CI | p value |
| --- | --- | --- | --- |
| LVEDV (per SD) | 1.60 | 1.26–2.04 | 0.0001 |
| Age (per SD) | 1.05 | 0.77–1.42 | 0.760 |
| Sex (male) | 0.91 | 0.47–1.74 | 0.780 |
| Frame rate (per SD) | 1.02 | 0.79–1.31 | 0.900 |
| Video duration (per SD) | 0.97 | 0.76–1.24 | 0.810 |

Cluster-robust standard errors were used to account for repeated studies within the same patient. Addition of acquisition variables did not materially alter the LVEDV association.

**Supplementary Table S3. Additional linked baseline characteristics by concordance status**

| Variable | Concordant | Discordant | p value |
| --- | --- | --- | --- |
| Hypertension, n (%) | 480 (49.3%) | 28 (58.3%) | 0.308 |
| Diabetes mellitus, n (%) | 234 (24.0%) | 16 (33.3%) | 0.242 |
| Atrial fibrillation, n (%) | 271 (27.8%) | 14 (29.2%) | 1.000 |
| Coronary artery disease, n (%) | 300 (30.8%) | 20 (41.7%) | 0.184 |
| Recorded heart failure diagnosis, n (%) | 306 (31.4%) | 20 (41.7%) | 0.222 |
| Body mass index (kg/m²), mean±SD | 29.5±6.7 | 30.1±7.8 | 0.645 |

**Supplementary Table S4. Clinically scaled interpretation of the LVEDV effect**

The primary model used LVEDV standardised to 1 cohort SD. To improve bedside interpretability, the same coefficient was rescaled to fixed volume increments using the observed cohort SD of 52.8 mL.

| Scaling unit | Approximate OR | Approximate 95% CI | Derivation |
| --- | --- | --- | --- |
| Per 1 SD (52.8 mL) | 1.61 | 1.27–2.05 | Primary fitted model |
| Per 10 mL | 1.09 | 1.05–1.15 | Coefficient rescaled from SD-based model |
| Per 50 mL | 1.57 | 1.25–1.97 | Coefficient rescaled from SD-based model |

These values are algebraic rescalings of the fitted SD-based coefficient; they do not represent a separate refitted model.

**Supplementary Table S5. Availability of selected structural measurements in the linked extraction**

| Measurement | Available studies | Concordant | Discordant | Comment |
| --- | --- | --- | --- | --- |
| IVSd / septal thickness | 668 / 1,022 | 1.1 ± 0.2 cm | 1.1 ± 0.3 cm | No between-group difference; p=0.436 |
| LVPW thickness | 0 / 1,022 | Not available | Not available | Structured field returned no usable records |
| LV mass | 0 / 1,022 | Not available | Not available | Structured field returned no usable records |

**Supplementary Figure S2. Representative A4C frame, exact ROI mask, and overlay**

This figure shows one study-level example from the credentialed dataset. The panel set illustrates the original A4C frame, the exact ROI mask available for the same study identifier, and the overlay used to visualise the segmentation-derived region. The present manuscript analysed the resulting structured labels; it did not perform new manual tracings.


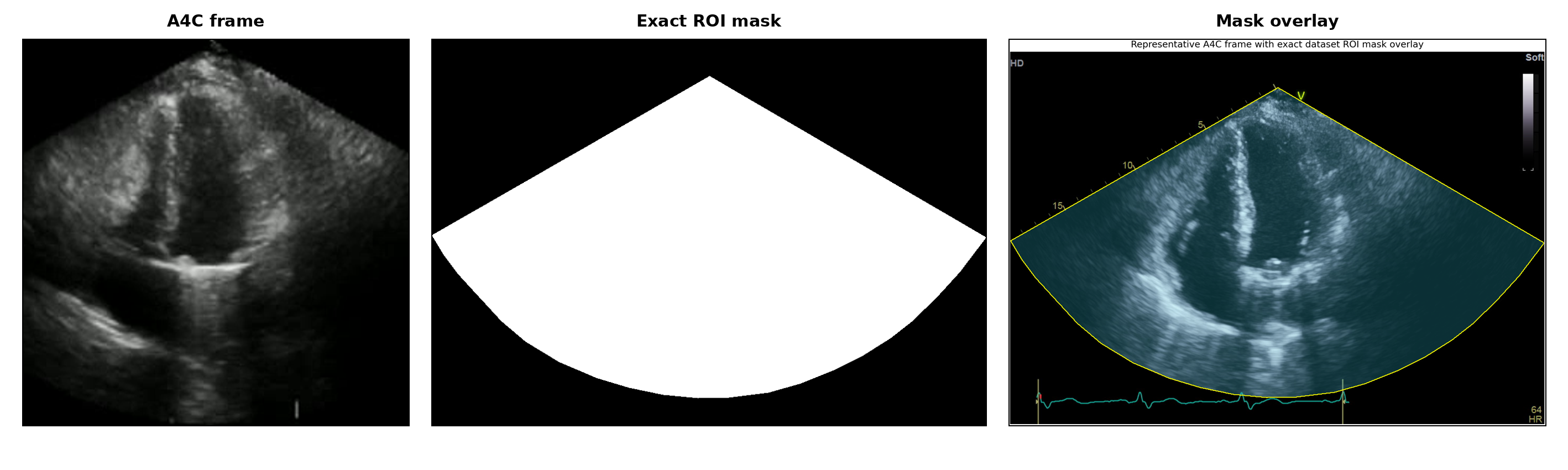

Supplement: Supplementary file 2 — Supplementary Material 2. [file 12872_2026_5952_MOESM2_ESM.docx]
